# Acceptability and preference of three inhalation devices assessed by the *Handling Questionnaire* in asthma and COPD patients

**DOI:** 10.1186/s40248-016-0044-5

**Published:** 2016-02-10

**Authors:** Roberto W. Dal Negro, Massimiliano Povero

**Affiliations:** 1 National Centre for Respiratory Phamacoeconomics & Pharmacoepidemiology, CESFAR, Verona, Italy; 2 AdRes Health Economics and Outcome Recourses, Torino, Italy

**Keywords:** Breezhaler, Bronchial Asthma, COPD, Handling Questionnaire, Genuair, Patient preference, Respimat

## Abstract

**Background:**

The patients’ criteria of preference for inhalation devices can affect the extent of their adherence to treatment and outcomes.

Aim of this study was to assess and compare the patients’ preference and acceptability (PPA) for Breezhaler and Genuair (both Dry Powder Inhalers), and for Respimat (a Soft Mist Inhaler) in asthma and COPD out-patients by means of the *Handling Questionnaire*.

**Methods:**

The *Handling Questionnaire* is a validated instrument which allows the investigation of different domains of PPA; it also takes into account the patients’ age and gender, together with their previous experience with the inhalation devices and their previous education approach to them. Differences in terms of preference, acceptance and usability were assessed by linear and logistic regressions in order to evaluate factors influencing the proper actuation.

**Results and Discussion:**

Data from 333 patients were collected: Genuair and Respimat were the most liked and perceived as the easiest to use at glance by patients, but also as the least problematic according to the patients’ and nurse’s judgments. Mean number of attempts for achieving the first effective actuation was the highest with Breezhaler (2.6 vs 1.6; *p* <0.0001). Linear regressions showed that longer the explanation, higher was the number of attempts to the first proper actuation (0.58 additional attempts every 10 s increase in the first explanation, *p* <0.0001). Devices requiring less manoeuvres for the actuation were used properly after less attempts (0.38 increase in the number of attempts every additional manoeuvres, *p* <0.0001). Respimat proved to be the most indicated in COPD patients since it was the most liked and its successful rate at first attempt was the highest. Logistic regressions confirmed these data. Previous experience with DPIs and/or MDIs did not affect the patient preference and acceptability, independently whether suffering from asthma or COPD.

**Conclusions:**

Substantial differences are existing in patient’s preference and acceptability for inhalation devices, mainly related to the handling and the understanding of the different devices.

## Background

Inhalation is the preferred route for delivering respiratory drugs (i.e., anti-asthma and chronic obstructive pulmonary disease - COPD), because therapeutic agents are delivered directly to the lungs, and offers a more rapid onset of action, thus allowing smaller doses, and a better efficacy-to-safety ratio compared to systemic options [1–4].

Devices for inhalation therapy represent crucial and critical factors which can substantially affect the therapeutic outcomes independently of the molecule used. Several factors related to the devices can, in fact, contribute to the effectiveness of treatment, such as their capability to consent the inhalation of a sufficient respirable fraction of drug (with a particle size ≤ 6 μ); reproducibility; precision; stability, and comfortable use, particularly in elderly [5–8].

Following that of Metered Dose Inhales (MSIs), the development of the Dry Powder Inhalers (DPIs) and of the Soft Mist Inhaler (SMIs) represented a milestone in the history of inhalation therapy as they do not contain propellants, generally minimize the variability of inhalation effectiveness due to the patient’s limits in cooperation and comprehension, and optimize the consistency of inhaled drug and the extent of its lung deposition [9, 10].

Nevertheless, the patient preference and acceptability (PPA) for inhaled devices still remain relevant points to investigate because their role is high indeed and ever increasing, and they can affect the extent of the patient’s adherence to inhalation therapy and then the outcomes of treatment [1, 11–13].

The PPA assessment is usually investigated by means of controlled and validated instruments (such as, questionnaires) [14–16]. The *Handling Questionnaire* is a validated questionnaire which was specifically designed to identify and compare the features for choice and acceptability of different inhalation devices in patients with persistent airway obstruction, namely bronchial asthma and chronic obstructive pulmonary disease (COPD) [14].

The aim of the present study was to assess and compare the PPA of three different Devices, two DPIs and one Soft Mist Inhaler (SMI), in out-patients with asthma and COPD by means of the *Handling Questionnaire*.

## Methods

PPA was measured for the three different devices: the DPIs Breezhaler and the Genuair and the Respimat SMI and they were labelled as A, B, and C. The *Handling Questionnaire* was the investigational tool used in the study [14]. The *Handling Questionnaire* is anonymous, it allows the investigation of different domains of PPA, and also takes into account the patients’ age and gender, together with their previous experience with inhalation devices and their previous education approach to them. The reason of including patients with and without previous experience and/or instruction to inhalation devices was to investigate how persistent or volatile is the effect of their instruction, and how this experience can make the difference in real life.

In the first step of the study, the functioning of each device was shown to each patient in random order by an expert nurse, institutionally and specifically involved in educational programs, and previously trained for the technical and the empathic approach to the study for three weeks. Then patients were required to report their choice simply stemming from their immediate perception, and also to specify the reason of their preference. In the second step (such as, after the careful nurse instruction of each device functioning) patients were required to prepare the actuation from each device by themselves, while the nurse was monitoring the patients’ technicality, noted their critical issues, counted the number of attempts needed for actuating the device effectively, and measured the time (in sec.) spent. Then, subjects were required to report their preference and specify the reason of their choice once experienced the device directly. To conclude this phase, the nurse added her comments for each device to those of each patient, in order to compare the two points of view at the end of the study. In the third step, patients were finally required to indicate their preference answering to ten closed questions pertaining to different aspects of acceptance and usability of each device.

As the three devices to compare were different in terms of number of actions needed for their actuation, they were presumed to be also different in terms of patients’ comprehension. Consequently, the time spent by the nurse to explain the correct functioning of each device to each patient up to three patient’s attempts was measured (in sec.) together with the corresponding time required to patients for practicing each device effectively up to three attempts.

The different characteristics of each device are reported in Table 1 together with the mean duration of the three nurse’s educational explanations and the corresponding patients’ operational attempts.

**Table 1:** Characteristics of devices tested in the questionnaire

Device name	Manoeuvres (n)^a^	Duration of nurse explanation (sec ± SD)			Time taken by patients to use the device (sec ± SD)		
		1^st^ att	2^nd^ att	3^rd^ att	1^st^ att	2^nd^ att	3^rd^ att
Breezhaler - A	7	60 ± 4	120 ± 5	150 ± 7	110 ± 4	130 ± 6	150 ± 8
Genuair - B	3	40 ± 3	60 ± 3	65 ± 5	40 ± 3	50 ± 4	60 ± 5
Respimat - C	4	50 ± 3	60 ± 4	90 ± 6	40 ± 3	50 ± 5	60 ± 4

*Att* attempt for the proper use of each device

^a^Number of manoeuvres needed to actuation

The differences between devices were assessed by using appropriate tests (Welch test for normal distributed variables, Wilcoxon test for not normal distributed variables, *χ*^2^ test and ANOVA test). All data are expressed as mean ± standard deviation (SD), or absolute numbers and percentage as appropriate. A *p* <0.05 was considered significant for all statistical tests. All analyses were performed using computer software R 3.1.2 [17].

Through a series of regressions, data collected from all tested devices were used for assessing the possible influence of the time spent by the nurse to explain the procedures, and the number of actions required to prepare the inhalation properly. More specifically, the number of attempts required before the proper use of each device was analysed by linear regression and the likelihood of success at the first attempt by logistic regression.

A subgroup analysis was also carried out according to patients’ original disease: bronchial asthma vs COPD, in order to test whether the respiratory condition could be associated to a different PPA for each device. Asthma patients were also included in the study in order to investigate if the preference, the handling and the usability of DPIs are disease-related or not. Moreover, we also decided to investigate if the understanding of asthma patients in the usability of some devices not yet allowed for their condition is different from that of COPD patients who are using this kind of device since long time.

## Results

The questionnaires from 333 consecutive patients were collected and analysed (47 % males, mean age 55 ± 18 years). The COPD/asthma ratio was 0.9 (i.e., 47 % COPD and 53 % asthma patients). Subjects proved well matched in terms of age and gender within the A, B, and C group.

More than half of patients had had previous experience or had been already instructed in using inhalation devices. No significant differences were detected among the three tested devices from this point of view (Table 2).

**Table 2:** Baseline characteristics for respondents in all and divided according to tested device

	All	Tested device			P
		A	B	C	
Mean age (SD)	55.2 ± 18.3	55.2 ± 18.3	54.8 ± 17.5	56.2 ± 18.3	0.99
Sex (% male)	46.5 %	46.5 %	49.4 %	45.3 %	0.99
Disease (% COPD)	47.4 %	47.4 %	47.7 %	46.9 %	>0.99
Previous experience with inhalation devices	63.7 %	63.7 %	66.7 %	63.2 %	0.98
Previous instruction to use of inhalation devices	60.7 %	60.7 %	64.1 %	61.6 %	>0.99

**Fig. 1:**
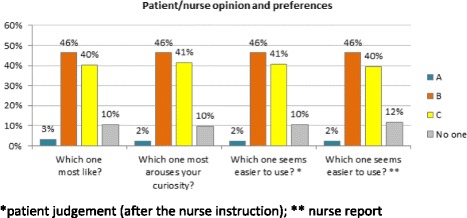
Results of preference questions: patient and nurse judgements

In general terms, devices B and C were the most liked; they were perceived as easier to use by the patients at glance, and the nurse’s judgement confirmed their perception (Fig. 1). From this point of view, the superiority of B and C was confirmed by pairwise comparisons reported in Table 3, and no significant differences were detected between devices B and C (p = ns).

**Table 3:**
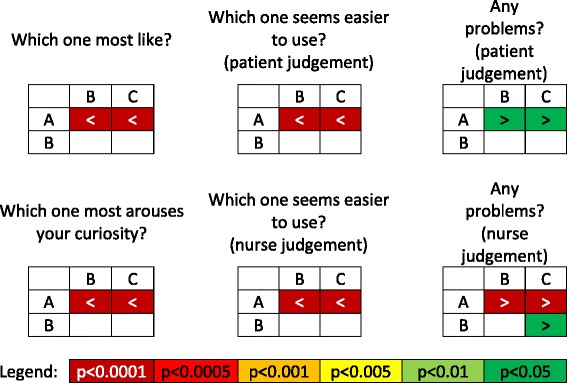
Pairwise comparison results, row vs column: “>” means that value measured for device in row is greater than value of device in column (vice versa for “<”); white cells represent comparisons that did not reach statistical significance (no differences are detected according to available data)

**Fig. 2:**
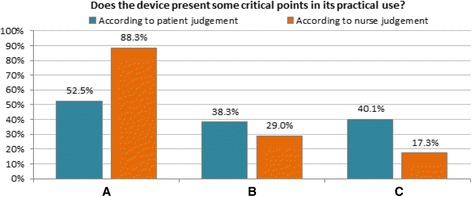
Presence of any problems found in the use of devices: patient’s vs nurse’s judgement

Devices B and C also proved less problematic according to both the patient’s and nurse’s judgement (Fig. 2); in particular, in pairwise comparisons of Table 3, device C had much less critical points than both A (*p* <0.0001) and B (*p* <0.05), according to the nurse’s report.

Moreover, patients seemed to underestimate the potential difficulties with the device A: actually, while approximately 50 % of patients claimed at glance (such as, during the nurse’s demonstration) some difficulties with device A, this proportion dramatically increased up to 90 % according to the nurse’s report when patients had to practice this device and prepare the actuation by themselves (Fig. 2). This information was achieved only because the study had been planned to compare the patients’ perception with the true judgment of the expert nurse who was always attending the effective patients’ procedure for actuation.

The number of attempts needed to patients for achieving the first proper actuation is a very important indicator for the true handling of devices in real life. The mean number of attempts for achieving the first effective actuation was 2.0 ± 1.1 in the whole all population, but devices B and C proved easier than A, and a lower mean number attempts were required (1.6 vs 2.6, *p* <0.0001). Furthermore, more than 80 % of patients were unable to use device A properly at the first attempt (Table 4).

**Table 4:** Measures of efficiency for each device

	All devices	A	B	C	*p*
N. attempts before achieving a proper actuation	2.0 ± 1.1	2.6 ± 1.1	1.6 ± 0.8	1.6 ± 1.0	<0.0001
Successful at 1st attempt (%)	42.6 %	18.0 %	55.7 %	62.4 %	<0.0001
N. attempts before achieving proper actuation (after failure of the first attempt)	2.8 ± 0.8	3.0 ± 0.8	2.4 ± 0.6	2.7 ± 1.0	<0.0001
Total time needed for a proper actuation^a^ (sec)	343 ± 308	615 ± 301	150 ± 95	170 ± 137	<0.0001

^a^Including time for nurse’s instruction and patients’ actuation

Also when only patients who failed the first attempt were considered, device B and C proved the best performer as they needed a less number attempts when compared to A (*p* <0.0001) (Table 4).

As both the nurse’s time for teaching the inhalation procedures and the patients’ time for preparing the actuation were shorter for devices B and C, total time spent in learning how to use these two devices properly was significantly lower than that spent for device A. In particular, the total time needed for the proper actuation was shorter using C vs A (170 vs 615 s., *p* <0.0001) (Table 4).

In general terms, a previous experience with whatever inhalation devices did not prove to reduce the number of attempts for achieving a proper actuation. In particular, patients with a previous experience in DPIs use required a slightly higher number of attempts than no-experienced subjects (2.1 vs 1.9, *p* <0.05). No statistical differences were detected with respect to previous experience with MDI.

Results of linear and logistic regressions agree perfectly (Table 5). Both the duration of the nurse’s explanation and the number of manoeuvres required proved statistically related to the number of patients’ attempts, and also to the probability of a successful actuation at the first attempt. In particular, longer the first and the second explanation (i.e., the very first one and those after the failure at the first patients’ attempt), higher the number of attempts: 0.58 and 0.26 additional attempts every 10 s. increase in explanation duration at the first and at the second instruction, respectively (*p* <0.0001). Conversely, longer the third explanation, lower the overall number of attempts (−0.37 attempts every additional 10 s, *p* <0.0001).

**Table 5:** Results of linear and logistic regressions performed to investigate the influence of devices characteristics on their practicality

All population	N. attempts before achieving correct preparation			Successful first attempt		
	delta	95 % C.I.	*p*-value	OR	95 % C.I.	*p*
Duration of first explanation	0.58^a^	0.33–0.82	*p* < 0.0001	0.45^a^	0.24–0.85	*p* < 0.05
Duration of second explanation	0.26^a^	0.19–0.33	*p* < 0.0001	0.58^a^	0.49–0.69	*p* < 0.0001
Duration of third explanation	−0.37^a^	−0.47–−0.27	*p* < 0.0001	1.94^a^	1.46–2.61	*p* < 0.0001
Number of manoeuvres	0.38^b^	0.32–0.43	*p* < 0.0001	0.50^b^	0.42–0.58	*p* < 0.0001

^a^Each 10 sec. increment in the duration of explanation, ^b^every additional manoeuvre to prepare the inhalation

Devices requiring less manoeuvres to set up the actuation proved to be used properly after less attempts: there is a 0.38 increase in the number of attempts every additional manoeuvres (*p* <0.0001).

Logistic regression on the probability of using the device properly at the first attempt confirms previous results; the increase/decrease is expressed in terms of OR (odds ratio) (Table 5).

### Subgroup analysis: asthma vs COPD patients

Subgroup analyses confirmed results obtained from the whole population: devices B and C were the preferred ones, and only mild differences were seen between asthma and COPD patients, probably due to some basic characteristics of the population sample. Actually, asthma patients were significantly younger than COPD patients (mean age 44 vs 68 years, *p* <0.0001), and less trained in the use of inhalation devices: they had less previous experience (56 vs 72 %, *p* <0.005) and less instruction in their use (55 vs 67 %, *p* <0.05).

Subgroup analysis showed a slight different trend between asthma and COPD patients. In general, asthma patients proved less difficulties in learning how to use the devices properly: the mean number of attempts was lower than in the COPD subjects: 1.9 vs 2.1, *p* <0.001. Also the successful rate at the first attempt was higher: 49 vs 36 %, *p* <0.0005, and the difference was mainly due to devices A and B. In particular, asthma patients preferred device B (50 % vs 34 % C, *p* <0.05), but both B and C seem indicated since their successful rate at the first attempt was high (67 and 64 %, respectively, *p* = ns).

Device C proved to be the most indicated in COPD patients since it was the most liked (47 %, p = NS) and its successful rate at first attempt was significantly higher than with both A (60 vs 11 %, *p* <0.0001) and B (60 vs 43 %, *p* <0.05) devices.

When the analysis was restricted only to patients that failed at the first attempt, no differences were detected between asthma and COPD patients (*p* >0.05).

According to these results, the mean time spent by each patient to learn how to use the devices properly was lower in the asthma group by almost 1 min (319 vs 371 s, *p* <0.005).

The use of the *Handling Questionnaire* showed that device A was the most critical and difficult to use: less than 3 % of patients liked this device at glance or perceived that it was easy to use. These results were confirmed by the nurse judgement, such as, more than 50 % of patients that tested the device A found difficulties in its practicality.

## Discussion

Even if inhalation represents the preferred route for delivering respiratory drugs, it is accepted since long time that the choice of the inhaler device to prescribe is usually empirically guided in real life, because almost completely independent of the knowledge of its technological characteristics and effective performance in the majority of cases [18–21].

Constant education should be essential for maintaining and progressively improving the patient’s confidence with prescribed device(s), but it is difficult to pursue because time consuming [22]. On the other hand, it has been shown that the skill in using inhalation devices is frequently inadequate among health care professionals (such as: GPs; medical students; pharmacists; nurses; respiratory physiotherapists, and even lung physicians) [13].

As the number of inhalation devices is continuously increasing and their specific characteristics can make the difference in their handling for the patients in real life, the criteria of choice related to the patients’ preference represent a crucial factor possibly affecting the treatment outcomes, in particular when the used device needs a complicated sequence of manoeuvres for its actuation, and the required procedures are not associated to a sufficient instruction degree of the patient [4, 23].

Furthermore, asthma and COPD patients are usually using their prescribed device(s) for long periods of time and then their acceptability and preference would represent a great issue, even if, unfortunately, the patient’s point of view was only infrequently regarded as a crucial variable which is able to influence the effectiveness of their treatment [18–21].

These aspects become even more important when we take into account that the molecules available on the market cannot be used with all devices interchangeably, because they are only delivered by a fixed device in the majority of cases.

Nevertheless, the ease of use and the understanding of the device actuation procedures represent a crucial point which can contribute to differentiate the patient’s preference and acceptability for devices different from MDIs, such as DPIs and MSIs. These differences could then represent a helpful indicator for grading the usability of each device in daily life [23, 24], particularly when the assessment of different domains which can influence the patient’s choice stems from comprehensive and validated investigational instruments, specifically designed [14–16, 22, 25–31].

Results from the present study point out to the real difference assessed by the *Handling Questionnaire* among the devices compared in terms of patient’s preference and acceptability. Respimat and Genuair resulted the most preferred, and those with the less difficulties in understanding the manoeuvres for actuating the inhalation and in practicing the inhalation effectively. Moreover, Respimat proved to be the easiest to use and the least problematic according to the nurse judgement (*p* <0.05) and the most easily learned, with a successful of more than 60 % rate at the first attempt. On the contrary, 80 % of patients were unable to use device A properly at the first attempt. This particular information points at the substantial discrepancy sometimes existing between the patients’ belief “at glance” and the handling of some devices in real life. It is a crucial issue because the effectiveness of inhalation treatments is highly depending on the proper use of devices, which sometimes are regarded in a too simplistic way by patients and doctors.

As partially expected, both the duration of the nurse’s explanation and the number of manoeuvres required proved statistically related to the number of patients’ attempts and also to the probability of a successful actuation at the first patient’s attempt.

To note that a previous generic experience with inhalation devices did not affect substantially the patient’s criteria of preference and acceptability. This evidence is plausible because no information is available on the quality of the instruction previously received by patients included in the present study.

Generally, devices requiring less manoeuvres to set up the actuation proved to be used properly after less attempts: there is a 0.38 increase in the number of attempts for every additional manoeuvre. Also the duration of the nurse’s instruction proved statistically related to the number of patients’ attempts and to the probability of a successful actuation at the first attempt.

In particular, subgroup analyses showed slight differences between asthma and COPD patients which are likely due to the fact that asthma subjects were significantly younger. Actually, asthma group proved to have less difficulty in learning how to use devices and they prove a higher successful rate at the first attempt than COPD patients. While COPD patients perceived the ease of use for B and C device equally, asthma patients preferred device B significantly.

The interest in patient’s preference revamped in recent years [22, 25–31] due to the increased awareness that PPA would contribute to a better adherence to the therapeutic strategy, thus leading to an increased effectiveness of treatment.

The preference for Respimat when compared to other devices (both MDIs and DPI) was already assessed in generic terms for COPD patients [25, 27], but the patient’s point of view was never compared with that of an expert nurse previously. Furthermore, for the first time in the present study the time spent for instruction and the number attempts for achieving the proper actuation were investigated analytically by linear and logistic regression in order to define the possible influence of the patient’s preference and acceptability.

Also comparison among Respimat vs Genuair and Breezhaler were never carried out previously at our knowledge, even if Breezhaler was proved to be much less preferred than Genuair in a recent study aimed to assess the patient’s satisfaction and the inhaler technique errors in COPD with these two devices [31].

## Conclusions

The ideal device is not existing yet, and the best one will likely be the next one. Nevertheless, substantial differences are existing among the available inhalation devices, both in terms of technical characteristics and of patient’s preference and acceptability.

For these reasons, a multidimensional approach to PPA was used in the present study, thus stemming from the patients’ perception; the patients’ motivated preference, and the patients’ degree of practical acceptability related to the technicalities required for actuation.

The investigation and the assessment of these differences should be pursued continuously by means of controlled and objective instrument of investigation. Those devices which prove as the most accepted by patients should be preferred in order to support the highest level of adherence to their respiratory treatment, particularly when long-lasting, as in patients with persistent airway obstruction.

As also the most effective inhalation treatment may not lead to a good disease control if the prescribed device is not accepted and properly used, the relevance of assessing PPA should be increasingly valued, because a correspondent higher convenience of best choices can also be presumed in terms of cost-effectiveness.
